# The utility of cardiovascular imaging in heart failure with preserved ejection fraction: diagnosis, biological classification and risk stratification

**DOI:** 10.1007/s10741-020-10047-9

**Published:** 2020-11-05

**Authors:** Gavin A. Lewis, Keith Pearce, Simon G. Williams, Erik B. Schelbert, Anita Macnab, Christopher A. Miller

**Affiliations:** 1 Division of Cardiovascular Sciences, School of Medical Sciences, Faculty of Biology, Medicine and Health, Manchester Academic Health Science Centre, University of Manchester, Oxford Road, Manchester, M13 9PL UK; 2 Manchester University NHS Foundation Trust, Southmoor Road, WythenshaweManchester, M23 9LT UK; 3 Department of Medicine, University of Pittsburgh School of Medicine, Pittsburgh, PA USA; 4 UPMC Cardiovascular Magnetic Resonance Center, Heart and Vascular Institute, Pittsburgh, PA USA; 5 Clinical and Translational Science Institute, University of Pittsburgh, Pittsburgh, PA USA; 6 Division of Cell-Matrix Biology & Regenerative Medicine, School of Biology, Faculty of Biology, Medicine & Health, Wellcome Centre for Cell-Matrix Research, Manchester Academic Health Science Centre, University of Manchester, Oxford Road, Manchester, M13 9PT UK

**Keywords:** Heart failure with preserved ejection fraction, Cardiovascular imaging, Diagnosis, Disease classification, Risk stratification

## Abstract

Heart failure with preserved ejection fraction (HFpEF) does not exist as a singular clinical or pathological entity but as a syndrome encompassing a wide range of clinical and biological phenotypes. There is an urgent need to progress from the unsuccessful ‘one-size-fits-all’ approach to more precise disease classification, in order to develop targeted therapies, personalise risk stratification and guide future research. In this regard, this review discusses the current and emerging roles of cardiovascular imaging for the diagnosis of HFpEF, for distilling HFpEF into distinct disease entities according to underlying pathobiology and for risk stratification.

## Introduction

Heart failure with preserved ejection fraction (HFpEF) does not exist as a singular entity but as a syndrome that encompasses a broad cohort of patients with a range of clinical and biological phenotypes [1, 2].

The utility of cardiovascular imaging in HFpEF serves three primary functions: (1) *diagnosis*: to determine whether patients’ symptoms and signs are due to heart failure (HF) and to identify specific causes of HF in the context of a normal or near normal left ventricular (LV) ejection fraction (EF); (2) *biological classification*: to characterise the underlying disease mechanisms; and (3) *risk stratification*: to guide prognosis.

The growing prevalence of HFpEF and its poor outcome dictate an urgent need for more precise disease classification in order to develop therapies that target specific pathophysiological mechanisms and to personalise risk stratification. The failure of multiple large phase III trials to identify an effective therapy serves to demonstrate that the “one-size fits all” approach to HFpEF is inadequate.

This review discusses the current and emerging roles of cardiac imaging for the diagnosis of HFpEF, for distilling HFpEF into distinct disease entities and for risk stratification.

## Diagnosis

### Diagnosing heart failure in the context of a preserved ejection fraction

Patients with suspected HFpEF represent a considerable diagnostic challenge, often having multiple co-morbid reasons for breathlessness, peripheral oedema and other clinical features consistent with HF. Whilst natriuretic peptides have increased diagnostic confidence for HF, they lack sensitivity and specificity in important subgroups such as obesity. Furthermore, their elevation may represent an advanced pathophysiological stage that may be less modifiable, where decompensation has already occurred [3, 4].

**Fig. 1:**
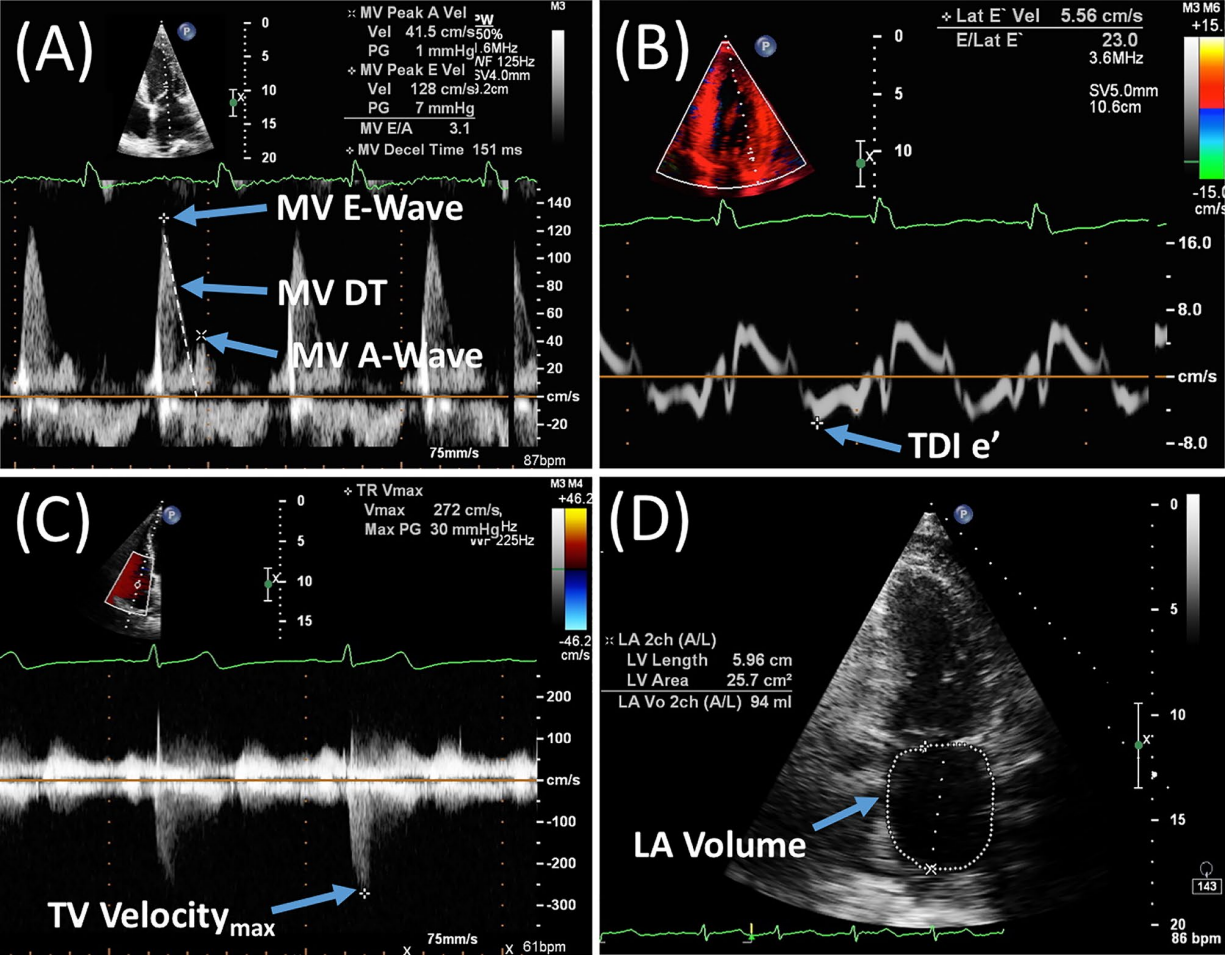
Echocardiographic measures used to assess diastolic dysfunction. **a** Mitral valve (MV) E-wave and A-wave peak velocities, obtained on pulse-wave Doppler, are combined to generate the E/A ratio. **b** Mitral valve E-wave peak velocity and tissue Doppler (TDI) *e*ʹ velocity (here measured at the lateral MV annulus; average *e*ʹ is calculated from both the lateral and medial sites) are combined to generate the *E*/*e*ʹ ratio. **c** Peak velocity of tricuspid valve (TV) regurgitation. **d** Apical 2-chamber view and measurement of left atrial (LA) volume. DT–deceleration time

Cardiac imaging, most commonly echocardiography, is employed in this context to investigate for evidence of LV ‘diastolic dysfunction’. LV diastolic dysfunction is caused by impaired LV relaxation and increased LV chamber stiffness. When diastolic dysfunction leads to impaired LV filling, LV diastolic pressure becomes elevated as a compensatory mechanism in order to maintain cardiac output. Echocardiographic assessment of LV diastolic function includes a number of indices that, when combined, provide an indication of LV filling and the sequelae of abnormal LV filling, particularly raised left atrial pressure (LAP). Specifically, the 2016 European Association of Cardiovascular Imaging (EACVI) and the American Society of Echocardiography (ASE) recommendations advise a combination of four variables for diagnosing diastolic dysfunction in patients with normal LV EF: annular *e*′ velocity (septal *e*′ < 7 cm/s, lateral *e*′ < 10 cm/s), average *E*/*e*′ ratio > 14, LA maximum volume index > 34 mL/m^2^ and peak tricuspid regurgitant velocity > 2.8 m/s [5]. Understanding the basis of non-invasive indices of diastolic function is crucial for their interpretation and for understanding their utility for identifying patients for inclusion in trials and for measuring the effect of interventions. Table 1 and Fig. 1 describe commonly used imaging measurements of diastolic function.

**Table 1:** Echocardiographic variables used to assess diastolic dysfunction and their association with invasive haemodynamic measurements

Variable of diastolic dysfunction	Background physiology	Dependent physiology	Surrogate measure	Evidence, calculations, and strength of correlation with surrogate measures
Peak E-wave velocity	Reflects the pressure gradient between the LA and LV after mitral valve opening during ‘early’ diastole, specifically the ‘rapid-filling’ phase	MV function, LA pressure and compliance, LV volume status (load), and LV relaxation	PCWP	*R* = 0.62 [77] *R* = 0.68 [78] *R* = 0.86 [79] *R* = 0.57 [80]
			Tau	No correlation demonstrated [81]
Peak A-wave velocity	Reflects the pressure gradient between the LA and LV during atrial contraction	LV relaxation and compliance (i.e. LA afterload), LA pressure, LA contractile function, and LA compliance	LVEDP	*R* = 0.85 (N.B. change in A-wave velocity with Valsalva) [82]
			PCWP	*R* = − 0.16 [77] *R* = 0.47 [79]
MV Deceleration time	Reflects the equalising of pressure between the LA and LV resulting in deceleration of ‘early’ MV flow	MV function, LV relaxation, LV chamber compliance and stiffness	PCWP	*R* = − 0.61 [83] *R* = − 0.55 [79] *R* = − 0.36 [80] *R* = − 0.07 [77]
			LVEDP	*R* = − 0.48 [83] *R* = 0.30 [84]
			LV chamber stiffness (*b*)	*R* = 0.39 [84] *R* = − 0.81 [85]
			LV stiffness constant (*β*)	*R* = 0.09 [84]
			LAP (direct)	*R* = − 0.73 [20]
			Tau	*R* = 0.28 [84] No correlation demonstrated [81]
			Pre-A-wave-LVDP	*R* = − 0.39 [86]
MV E/A ratio	Non-physiological (a method for identifying descriptive ‘filling patterns’, i.e. normal, impaired relaxation, pseudonormalisation, restrictive)	(As for peak E-wave and peak A-wave velocity)	LAP (direct)	*R* = 0.49 [20]
			mLVDP	*R* = 0.59 [18]
			LVEDP	*R* = − 0.04 [84] No correlation reported [82]
			PCWP	*R* = 0.42 [79] *R* = 0.46 [80] *R* = 0.52 [77] *R* = 0.63 [83]
			Tau	*R* = − 0.46 [87] *R* = − 0.36 [84]
			LV chamber stiffness (*b*)	*R* = − 0.24 [84]
			LV stiffness constant (*β*)	*R* = − 0.22 [84]
TDI *e*ʹ velocity	Reflects myocardial fibre lengthening during LV relaxation as a measurement of MV annular motion during early diastole	LV relaxation, elastic restoring forces, and load	PCWP	*R* = − 0.37 [80] *R* = 0.13 [78] *R* = 0.81 (N.B. medial annulus) [79]
			mLVDP	*R* = 0.36 (N.B. medial annulus) [18]
			Tau	*R* = − 0.81 (N.B. lateral annulus) [81] *R* = − 0.70 [12] *R* = − 0.56 [87] *R* = − 0.51 [88] *R* = − 0.46 [18] *R* = − 0.33 [84]
			LVEDP	*R* = − 0.50 [84] *R* = 0.61 [88]
			LV chamber stiffness (b)	*R* = − 0.39 [84]
			LV stiffness constant (*β*)	*R* = − 0.41 [84]
TDI *E*/*e*ʹ ratio	Non-physiological (peak E-wave velocity corrected for the influence of myocardial relaxation by dividing by TDI *e*ʹ velocity)	As per peak E-wave velocity and TDI *e*ʹ velocity	PCWP	*R* = 0.17 (N.B. lateral annulus) [79] *R* = 0.63 (N.B. medial annulus) [26] *R* = 0.86; PCWP = [(1.47 × *E*/*e*ʹ) + 1.55] [80] *R* = 0.87; PCWP = [(1.24 × *E*/*e*ʹ) + 1.9] [78]
			mLVDP	*R* = 0.64 (N.B. medial annulus) [18]
			pre-A-wave-LVDP	*R* = 0.74 [86]
			LVEDP	*R* = 0.71 [84] *R* = 0.79 [88] LVEDP = [(0.85 × *E*/*e*ʹ) + 4.4] [89]
			Tau	*R* = 0.34 [84]
			LV chamber stiffness (b)	*R* = 0.46; *b* = [(0.016 × *E*/*e*ʹ) + 0.1] [84]
			LV stiffness constant (*β*)	*R* = 0.53; *β* = [(0.002 × *E*/*e*ʹ) + 0.008] [84]
Peak TR-velocity	Reflects the pressure gradient between the RV and RA during systole	TV function (presence of TR), RA pressure, PA pressure and compliance	Estimated PCWP	*R* = 0.73 (N.B. correlation with PASP) [90]
			TR velocity > 2.8 m/s (ASE/EACI criteria for diastolic dysfunction)	A TR velocity cut-off of > 2.8 m/s is based on population studies of upper limits of normal, endorsed by international guidelines [91, 92]
LA volume index	Reflects chronic LA pressure elevation, manifesting as increased LA volume (indexed for BSA)	LA stiffness, volume status (load), MV function	LA volume index > 34 mL/m^2^ (ASE/EACI criteria for diastolic dysfunction)	Tsang 2002 et al. [93] defined an LA volume index cut off > 34 mL/m^2^ as a predictor of *E*/*e*ʹ > 15 (sensitivity 86%, specificity 66%). ‘Moderate’ LA dilatation defined in ASE 2005 guidelines [94], based on population studies. Described in initial grading system for diastolic dysfunction by Nagueh et al. [95]. Revised in updated ASE 2015 guidelines to define 34mls/m2 as ULN for LA size [96]

*ASE* American Society of Echocardiography; *BSA* body surface area; *EACI* European Association of Cardiovascular Imaging; *LA* left atrium; *LAP* left atrial pressure; *LV* left ventricle; *LVDP* left ventricular diastolic pressure; *LVEDP* left ventricular end-diastolic pressure; *mLVDP* mean left ventricular diastolic pressure; *MV* mitral valve; *PA* pulmonary artery; *PASP* pulmonary artery systolic pressure; *PCWP* pulmonary capillary wedge pressure; *RA* right atrium; *RV* right ventricle; *TDI* tissue Doppler imaging; *TR* pulmonary tricuspid regurgitation; *ULN* upper limit of normal

It is important to recognise that diastolic dysfunction does not equate to a diagnosis of HF, rather a mechanism for its presence; indeed, it may identify an earlier stage of HF than natriuretic peptides [6]. It is also important to note that across HFpEF trials, a third of patients consistently have normal diastolic function, suggesting that diastolic dysfunction is not the causative mechanism in a significant proportion of patients [2].

### LV filling

LV filling pressure refers to the pressure difference between the left atrium (LA) and LV that is responsible for LV filling during diastole. In the healthy heart at end-diastole, pulmonary artery pressure (PAP), LAP and LV end-diastolic pressures (LVEDP) effectively equalise and can collectively be referred to as ‘LV filling pressures’ [7]. However, in disease, this equilibrium is disturbed in an unpredictable manner and thus whilst widely used, the term ‘LV filling pressures’ is ambiguous and can be misleading. Studies generally include one of these measurements to indicate LV filling pressure, the choice of which depends upon what is available and the purpose of the study (idealistically LAP when the focus is pulmonary congestion and LVEDP when the focus is myocardial mechanical function). Indeed, the variable strength of association between echocardiographic diastolic indices and invasive haemodynamic measurements in part reflects the use of different invasive correlates (Table 1).

LV filling is determined by ventricular relaxation and chamber compliance [8]. Ventricular relaxation, defined by the rate and duration of LV pressure decay, begins in mid-systole and ends in early diastole and is an active process related to myofibrillar dissociation and calcium reuptake [9]. A number of mechanisms contribute to dysfunctional ventricular relaxation, including delayed inactivation, reduced restoring forces, diminished load dependence, increased mechanical non-uniformity of relaxation and the inability to increase relaxation rate in response to exercise (reduced diastolic reserve) [10]. The time constant (*tau*) of the isovolumetric fall in LV pressure is considered the gold standard invasive measurement of LV relaxation (Table 2).

**Table 2:** Invasive haemodynamic measurements of left ventricular (LV) stiffness and LV relaxation

Invasive haemodynamic measure	Physiological relevance	Definition(s)	Calculation
LV stiffness constant (mL^−1^/mL) (denoted as *β;* also referred to as *passive LV stiffness constant*)	Stiffness is the change in ventricular pressure relative to a change in volume of the ventricular chamber (dP/dV). The relationship is non-linear	Multiple pressure–volume curves generated during preload reduction generates the EDPVR (Fig. 4). EDPVR is exponential, therefore an exponential or power-function curve-fit equation is used	*P* = αe^*β* V^ or *P* = αV^*β*^ P = pressure; V = volume; both α and *β* are curve fitting constants
LV chamber stiffness (mmHg/mL) (denoted as* b* or *dP/dV*; also referred to as *LV operating stiffness*, denoted by *K*_LV_)	Stiffness is the change in ventricular pressure relative to a change in volume of the ventricular chamber (dP/dV)	The slope of LV pressure change relative to LV volume change. LV pressure change defined as *difference between LV minimal pressure and LVEDP*	*b* = dP/dV
Tau (ms) (denoted as τ; also referred to as *time constant of LV relaxation*)	A measure of ventricular relaxation during a defined isovolumetric period. The most commonly used isovolumetric relaxation period is defined as the time from dP/dt_min_ to ‘Tau end-point’, defined as the time when LV pressure falls to 5 mmHg above LVEDP of next cardiac cycle (to ensure it occurs before MV opening) [97]	Weiss et al. [98] (zero-asymptote)	*P*(*t*) = *P*_0_ × *e*^−t/τ^ *P*(*t*) = LVP as a function of time; *P*_0_ = pressure at dP/dt_min_ when *t* = 0 ms
		Raff et al. [97] (non-zero asymptote)	*P*(*t*) = (*P*_0_-*P*_∞_) × *e*^−t/τ^ + *P*_∞_ *P*(*t*) = LVP as a function of time; *P*_0_ = pressure at dP/dt_min_ when *t* = 0 ms; *P*_∞_ = baseline (asymptotic) pressure
		Half-pressure method (Tau ½): time required for LV pressure to decline to half the value recorded at dP/dt_min_ [9]	

*EDPVR* end-diastolic pressure-volume relationship; *LVP* left ventricular pressure; *MV* mitral valve

**Fig. 2:**
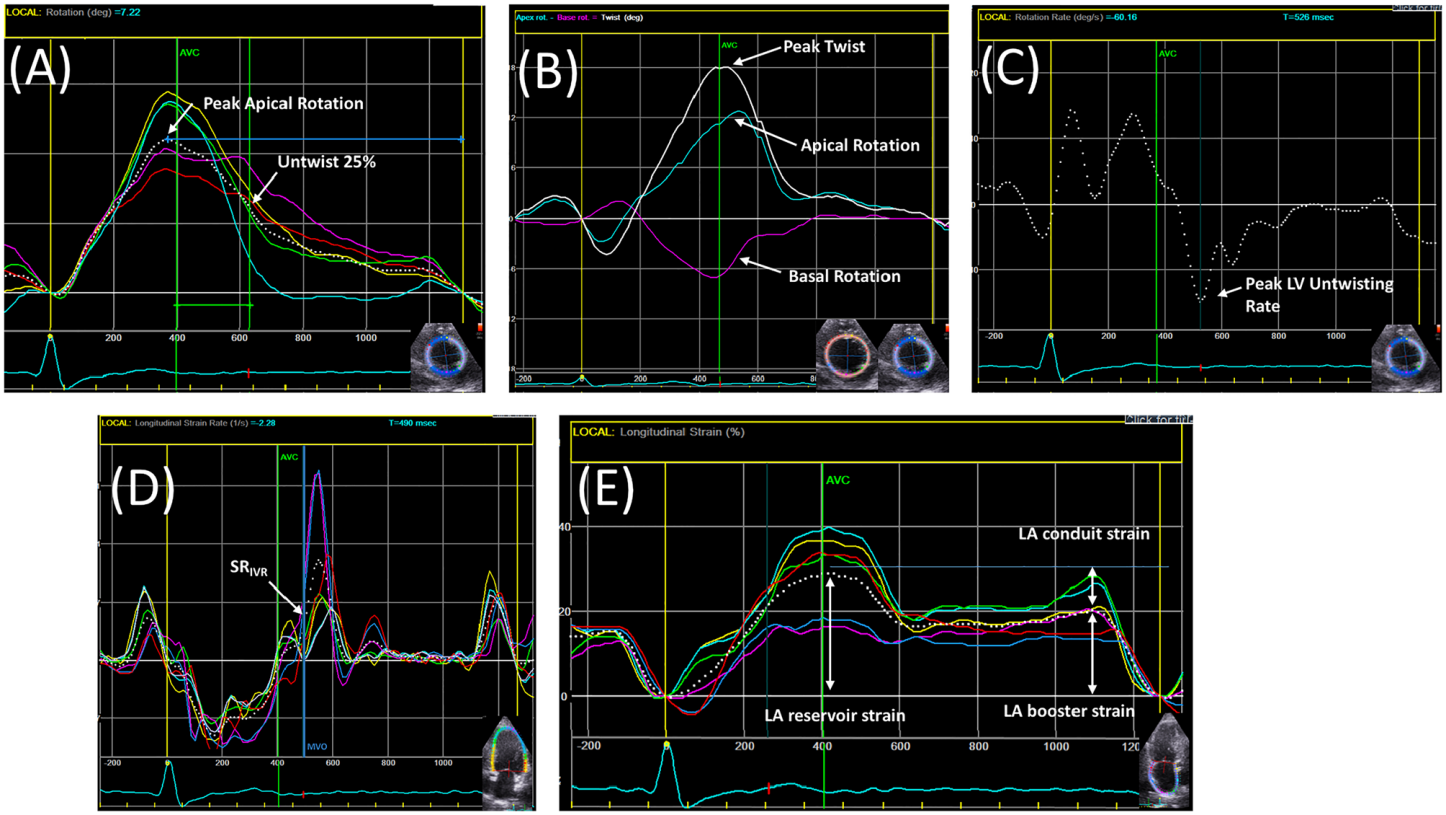
Speckle-tracking measures on echocardiography. **a** Peak apical rotation and untwist measurements from the apical short-axis view (SHAX). **b** Basal rotation and apical rotation generates peak left ventricular (LV) twist, measured from the basal-SHAX and apical-SHAX, respectively. **c** Peak LV untwisting rate, measured from the apical-SHAX. **d** Peak global longitudinal strain rate during isovolumetric relaxation (SR_IVR_). **e** Left atrial (LA) strain in the apical 4-chamber view measured as reservoir, conduit, and booster strain. AVC–aortic valve closure; MVO–mitral valve opening

Early diastolic mitral annular velocity (*e*′), measured using tissue Doppler imaging (TDI), provides a reflection of myocardial fibre lengthening and is used to assess ventricular relaxation; however, *e*′ is also determined by other factors, including elastic restoring forces, ventricular loading and acquisition angle, and as a result, the association between *e*′ and *tau* is variable (Table 1). A number of novel echocardiographic indices of ventricular relaxation have been proposed. Peak global longitudinal diastolic strain rate during isovolumetric relaxation (SR_IVR_), measured with speckle-tracking, is a potentially load-independent measure of global ventricular relaxation and shows a moderately strong inverse correlation with *tau* (*r* = − 0.74), although it is image quality-dependent (Fig. 2) [11]. Peak reverse ejection intraventricular pressure difference, measured using colour M-mode Doppler, also shows a moderately strong inverse correlation with *tau* (*r* = − 0.71), which improves when combined with *e*′ (*r* = − 0.84), and colour M-mode Doppler flow propagation velocity (*V*_p_) has shown strong inverse correlation with *tau* (*r* = − 0.75), although neither method measures myocardial deformation directly and both are technically demanding [12, 13].

LV untwisting during isovolumetric relaxation releases energy stored during systolic twisting in the extracellular matrix (ECM) and in the sarcomeric macromolecule titin and is purported as a measurement of LV relaxation. Peak LV untwisting rate has shown load-independent associations with *tau* in preclinical studies [14, 15].

Effective LV chamber compliance is defined as change in volume over change in pressure and is conceptualised as a passive process related to viscoelastic properties of the myocardium, which are governed by factors such as aging, the ECM and titin. Broader LV compliance is determined by additional factors such as the pericardium and ventricular interaction. Chamber stiffness is the reciprocal of chamber compliance and can be measured invasively in a number of ways (Table 2). The end-diastolic pressure–volume relationship (EDPVR) is exponential and measures LV chamber stiffness from multiple measurements of LVEDP and LV end-diastolic volume (EDV). A curve-fit equation (most commonly a power function or exponential equation) generates *α* (a curve-fitting constant) and *β* (the LV ‘stiffness constant’), the latter of which is influenced by geometrical changes, such as LV hypertrophy, and intrinsic myocardial stiffness. Using this equation, an echocardiographic method has been developed to estimate single-beat *β*, in which LVEDP is estimated from *E*/*e*′. To account for covariance between *α* and *β*, calculated LV EDV at an idealised LVEDP (e.g. 20 mmHg, denoted by EDV_20_) can compare LV stiffness between groups in cohort studies [16]. In a large prospective observational study (*n* = 419), in which patients with HFpEF were enrolled following HF hospitalisation, decreased LV compliance, indicated by reduced EDV_20_, was independently associated with HF hospitalisation (adjusted hazard ratio (HR) 1.67 [confidence interval: 1.22–2.30]) and combined cardiovascular hospitalisation and/or death (adjusted HR 1.39 [confidence interval: 1.10–1.75]) in multivariable analysis [17].

Rather than evaluating its constituent components, LV filling is usually assessed more broadly using mitral inflow indices such as E/A ratio and E wave velocity deceleration time (Table 1). However, as is well recognised, mitral E/A ratio has a U-shaped relationship with LVEDP, making it difficult to differentiate normal from abnormal (‘pseudo-normalisation’), particularly in the setting of normal LVEF, and mitral E wave deceleration time does not relate to LVEDP when LVEF is normal [18].

### LA pressure

Raised LAP is the key marker for identifying cardiac-induced pulmonary venous congestion [7]. LAP is rarely measured directly; instead, a number of invasive surrogates have historically been used, including pulmonary capillary wedge pressure (PCWP), pre-*a-*wave LV pressure (pre-a-LVP), mean LV diastolic pressure (mLVDP) and LVEDP. Each of these measurements shows a strong correlation with LAP in health, but in disease, the relationships are more variable. For example, LVEDP can be elevated without an increase in LAP, which is important when considering the validity of non-invasive assessments [18–20].

There have been many attempts to identify a non-invasive measurement of LAP; however, all are influenced by factors other than LAP. LA volume is used as a relatively straightforward assessment of LAP; however, it is neither sensitive nor specific. One-third to one-half of patients in HFpEF studies have normal LA size and the relationship between LA size and outcome is variable [21, 22]. In the echocardiographic substudy of TOPCAT (Treatment of Preserved Cardiac Function Heart Failure With an Aldosterone Antagonist Trial), patients enrolled on the basis of elevated natriuretic peptides did have larger LA volumes than those enrolled on the basis of previous hospitalization, but median LA volume was lower than the EACVI/ASE threshold for diastolic dysfunction (30.3 [23.0–38.9] mL/m^2^) [22]. Borlaug et al. [23] showed that a third of patients with HFpEF, diagnosed using invasive haemodynamic measurements made during exercise (exercise PCWP ≥ 25 mmHg), had normal LA size. Furthermore, in around a third of patients with HFpEF included in a comprehensive phenotyping study by Shah et al. [24], LA volume was lower than the EACVI/ASE threshold despite PCWP being elevated (19.0 ± 6.3 mmHg). Conversely, one-third of patients with hypertension without HF have LA enlargement, and half of patients with normal resting and exercise PCWP have LA dilatation [25, 26].

The *E*/*e*′ ratio (Table 1) was developed in order to correct mitral E-wave velocity, which reflects the LA-LV pressure gradient during early diastole and is dependent on the rate of LV relaxation and LAP as well as a number of confounding factors, for LV relaxation in order to provide a more discriminatory indication of LAP, and hence differentiate normal from pseudo-normal mitral inflow. Indeed, studies do generally agree that a markedly elevated *E*/*e*′ ratio (> 13–15) is highly specific for increased ‘LV filling pressures’ and a ratio of < 8 usually indicates normal ‘LV filling pressures’ [18]. However, *E*/*e*′ ratio is influenced by mitral valve and pericardial disease and there remains a wide grey-zone where it is unhelpful. In patients with preserved LVEF (> 50%), *E*/*e*′ ratio does associate with mLVDP more closely than other echocardiographic indices; however, the relationship is modest (*r* = 0.47 in the study by Ommen et al.[18]), and much less strong than in patients with reduced LVEF [8].

### Exercise

It is increasingly recognised that LAP may only become elevated during exercise [26, 27]. This group of patients, which describe exertional dyspnoea but have normal resting diastolic function and normal natriuretic peptides, represent a particular diagnostic challenge.

In the healthy heart, both early diastolic suction (increasing mitral valve flow) and myocardial relaxation are augmented during exercise; thus, *E*/*e*′ ratio is preserved. In a group of 74 consecutive patients with EF ≥ 50% referred for haemodynamic investigations for exertional dyspnoea, elevated PCWP (≥ 25 mmHg) was evident during exercise in around a third of patients [26]. Addition of exercise *E*/*e*′ to resting *E*/*e*′ improved sensitivity for identifying patients with HFpEF to 90%, albeit the cost of reduced specificity (75%).

Current guidelines recommend exercise echocardiography in patients with indeterminate resting measurements (i.e. grey-zone *E*/*e*′) and in patients with diagnostic uncertainty [5]. Recently, LA strain measured using speckle-tracking echocardiography *at rest* has shown comparable sensitivity and specificity (86% and 79%, respectively) for identifying elevated exercise PCWP [28]. The utility of exercise LA strain is yet to be determined.

### Specific causes of HFpEF

**Fig. 3:**
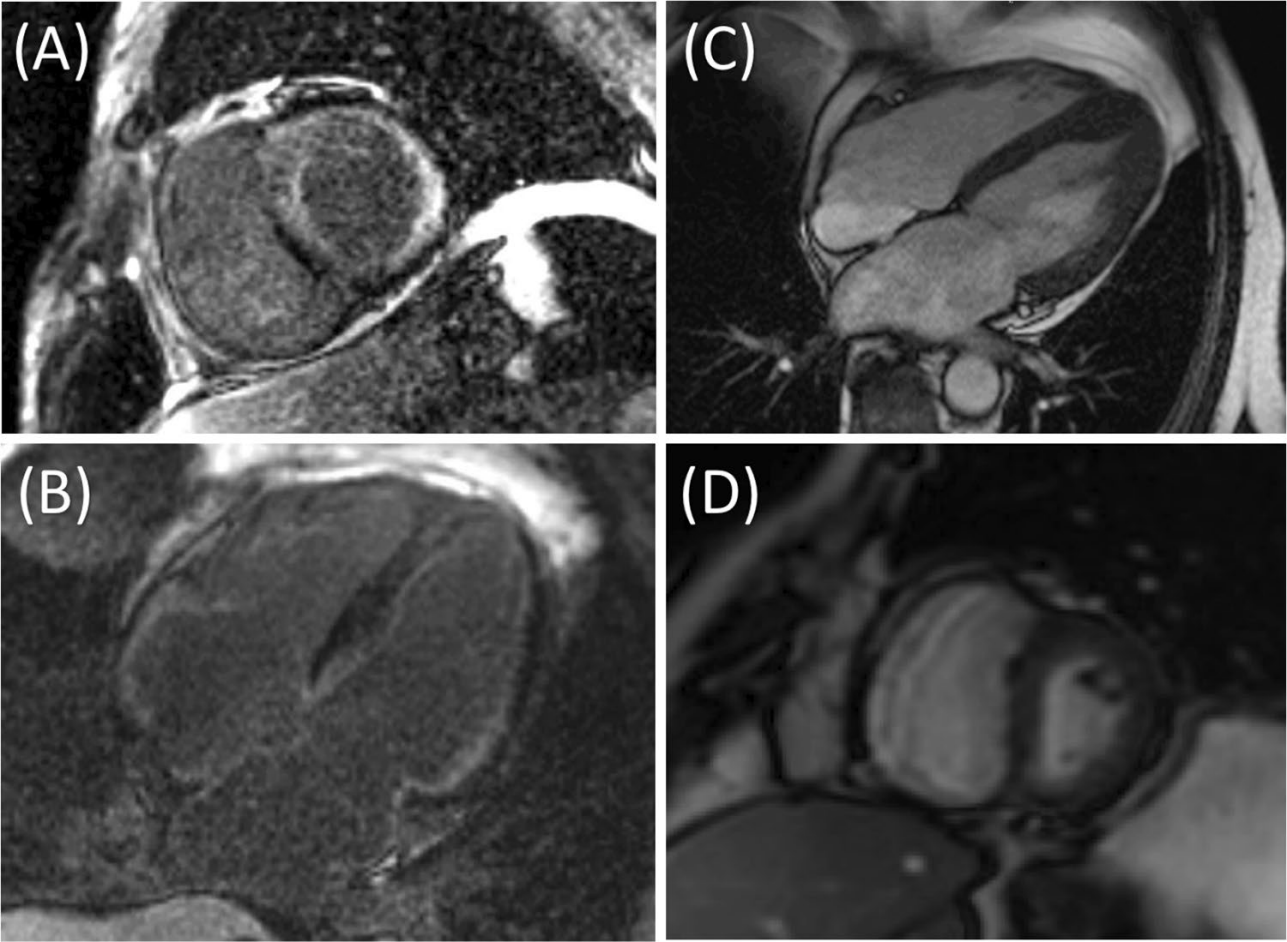
Specific causes of heart failure with preserved ejection fraction (HFpEF) diagnosed on cardiac magnetic resonance imaging (CMR). **a** Short axis late-gadolinium enhancement (LGE) sequence in a patient with HFpEF secondary to cardiac amyloidosis. Diffuse subendocardial enhancement is seen in the left and right ventricles. **b** Apical 4-chamber view in the same patient showing subendocardial LGE. **c** Apical 4-chamber cine in a patient initially diagnosed with ‘HFpEF’. Increased wall thickening and hypertrophy are seen at the left ventricular (LV) apex typical of apical hypertrophic cardiomyopathy. **d** Short axis cine in a patient presenting with ‘HFpEF’, demonstrating pericardial thickening and ‘D’ shaped flattening of the septum towards the LV during diastole. Subsequently diagnosed with pericardial constriction

There are a number of specific causes of HF in the context of a normal EF (e.g. amyloid, hypertrophic cardiomyopathy, Fabry disease etc.) that potentially account for as many as 25% of patients with a label of ‘HFpEF’ (Fig. 3) [29]. This has important implications both for clinical trials, where their inclusion may negate the beneficial effect of the intervention (as cited in the recent prospective comparison of angiotensin receptor-neprilysin inhibitor with angiotensin-receptor blockers Global Outcomes in HF with Preserved Ejection Fraction (PARAGON-HF) trial [30]), and clinical management, where specific therapies are available (e.g. light-chain amyloidosis). Incorporating advanced imaging, such as cardiovascular magnetic resonance (CMR) imaging, into HFpEF trial recruitment, at least for patients displaying notable characteristics such as very high natriuretic peptide levels, may be beneficial.

## Biological classification

HFpEF involves multiple pathophysiological mechanisms, which drive the heterogeneous phenotypes that are evident clinically [2]. By characterising these biological processes, cardiovascular imaging has the potential to distil HFpEF into distinct disease entities, which may provide more precise risk stratification and identify individual patients for targeted interventions.

### Myocardial fibrosis

**Fig. 4:**
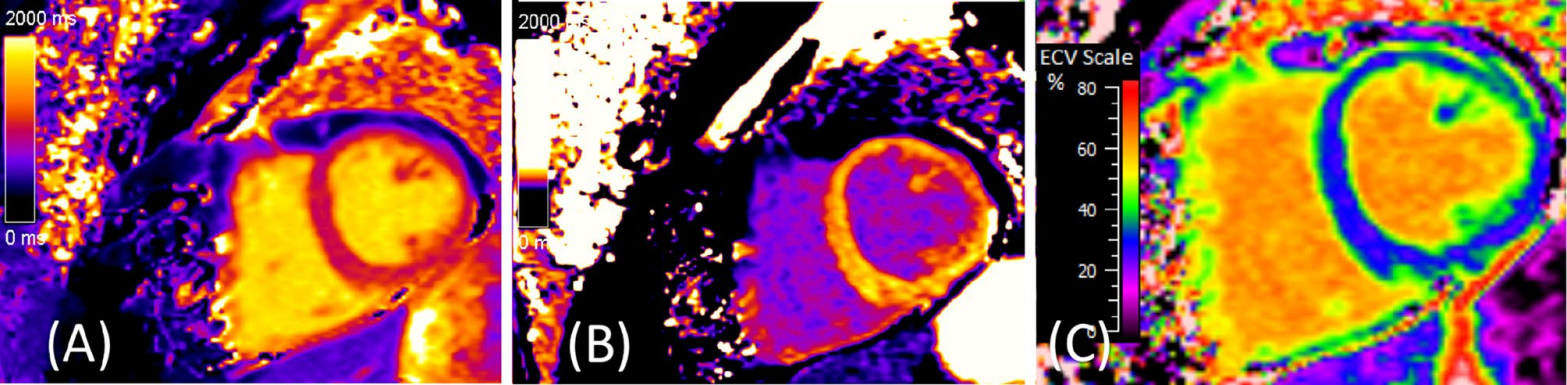
Myocardial fibrosis assessed by T1-mapping and extracellular volume (ECV). **a** Native T1 map of the basal left ventricle (native T1 time 1021 ms). **b** Post-contrast T1 map (post-contrast T1 time 432 ms). **c** ECV map (calculated ECV 32.6%)

ECM expansion secondary to collagen accumulation is associated with increased myocardial stiffness [31, 32]. Extracellular volume fraction (ECV), measured using CMR, provides a non-invasive measurement of myocardial fibrosis **(**Fig. 4**)** [33]. Elevated ECV is common in HFpEF but not universal; Schelbert et al. [34] showed that at least a quarter of patients diagnosed with HFpEF have an ECV less than the median value of a non-HFpEF control group. Furthermore, elevated ECV can be seen in hypertensive patients without HF [35]. As such, while ECV should not be regarded as a diagnostic biomarker for ‘HFpEF’ as a single entity, it can be used to identify a subgroup of patients with a fibrotic phenotype.

Rommel et al. [36] demonstrated a strong correlation between ECV and invasively measured LV stiffness constant *β* (*r* = 0.75, *p* < 0.01), and ECV was the only independent predictor of *β* on multivariable analysis. To illustrate the potential utility of ECV for identifying phenotypic subtypes of HFpEF, when patients in the study by Rommel et al. were dichotomized according to median ECV, both groups showed a pathological upward shift of the end-diastolic pressure–volume relationship during exercise; however, the dominant pathophysiology was an increase in myocardial passive stiffness in patients with elevated ECM volume, whereas the dominant mechanisms were arterial stiffness and impaired active relaxation in patients with a below-median ECM volume.

Importantly, myocardial fibrosis, measured using ECV, strongly associates with adverse outcome in patients with HFpEF, including death and hospitalisation for HF [34], and as such, myocardial fibrosis is identified as a potentially important therapeutic target.

### Impaired myocardial energetics

**Fig. 5:**
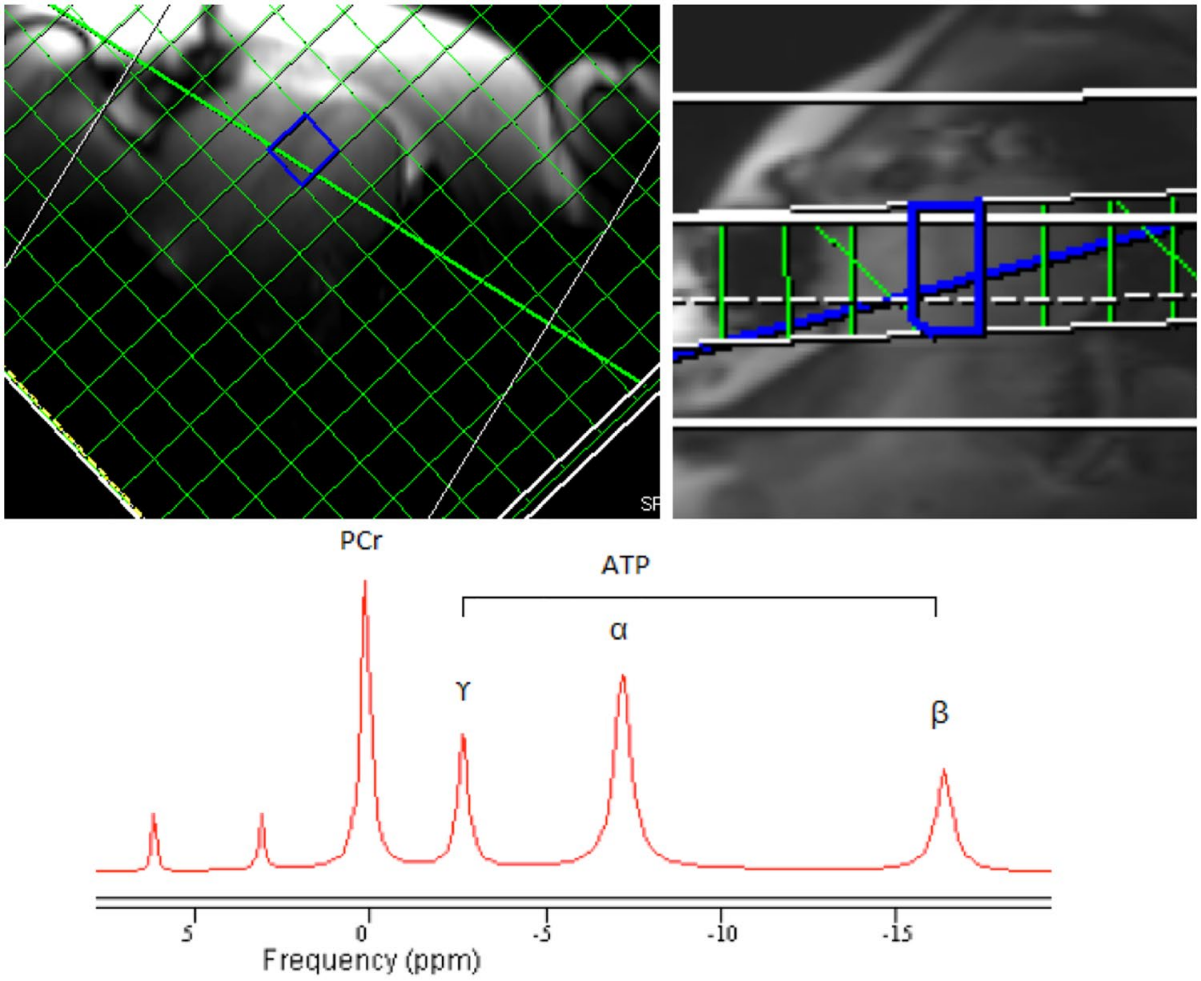
Myocardial energetics assessed by ^31^phosphorous-magnetic resonance spectroscopy (^31^P-MRS). A chemical-shift imaging ^31^P-MRS sequence, with voxel grid aligned to the ventricular septum, generates a frequency spectrum denoting phosphocreatine (PCr), γ-, α- and β-adenosine triphosphate (ATP), thus calculating the PCr to ATP ratio

Active LV relaxation, occurring in late systole and early diastole, is an adenosine triphosphate (ATP)-consuming process. Metabolomic profiling has demonstrated dysfunctional fatty acid oxidation in HFpEF [37], and myocardial fibrosis and microvascular dysfunction potentially render cardiomyocytes prone to hypoxia [38]. Phosphorous-31 magnetic resonance spectroscopy (^31^P-MRS) can non-invasively quantify the phosphocreatine (PCr) to ATP ratio, an index of energetic status that reflects the creatine kinase energy shuttle (Fig. 5) [39]. Phan et al. [40] showed HFpEF to be associated with significantly reduced resting PCr to ATP ratio compared with healthy controls (1.57 ± 0.52 and 2.14 ± 0.63 respectively, *p* = 0.003) and prolonged time to peak diastolic filling (a measure of active relaxation) during exercise. Proton magnetic resonance spectroscopy (^1^H-MRS) provides non-invasive measurement of myocardial triglyceride content and the identification of myocardial steatosis. Mahmod et al. [41], who measured both ^31^P-MRS and ^1^H-MRS, demonstrated significantly reduced PCr to ATP ratio and greater MTG in patients with HFpEF compared with healthy controls, with triglyceride content (but not PCr to ATP ratio) being independently associated with diastolic strain on multivariable analysis. The on-going phase 2 Pirfenidone in Heart Failure with Preserved Ejection Fraction (PIROUETTE) trial [42] includes assessment of the relationship between myocardial fibrosis and energetics, measured using ^31^P-MRS, and the impact of an antifibrotic intervention.

### Myocardial microvascular dysfunction

Invasive studies have demonstrated reduced myocardial oxygen delivery during exercise in a small group of HFpEF patients (*n* = 9) compared with hypertensive and normotensive controls [43]. In the absence of significant epicardial coronary artery disease, such findings likely reflect microvascular dysfunction, which may occur due to impaired endothelial function and blunted response to local vasoactive mediators (e.g. nitric oxide) [44]. Non-invasive assessment of coronary flow reserve (CFR) has been performed with Rubidium-82 cardiac positron emission tomography (PET) and phase-contrast CMR coronary sinus blood flow, with both techniques demonstrating reduced CFR on pharmacological stress in HFpEF patients compared with hypertensive and healthy controls [45, 46]. In a cohort of patients (*n* = 201) without significant epicardial coronary artery disease undergoing clinically indicated Rubidium-82 PET, Taqueti et al. [47] showed that a CFR of < 2.0 was associated with an increased risk of incident HFpEF hospitalisation on multivariable analysis (HR 2.47, adjusted for estimated glomerular filtration rate (eGFR), *E*/*e*′, LVEF, troponin and history of atrial fibrillation (AF)).

The PROMIS-HFpEF study [48] measured CRF in 202 HFpEF patients using echocardiographic pulse-wave Doppler of the left anterior descending artery via a modified 2-chamber apical view at rest and during adenosine stress, a method previously shown to be reproducible and demonstrate good agreement with cardiac PET-measured CFR [49]. Coronary microvascular dysfunction (defined as CFR < 2.5) was highly prevalent, seen in 75% of patients, and a CFR < 2.5 was associated with elevated N-terminal pro-brain natriuretic peptide (NTproBNP) and lower reactive hyperaemic index (a measure of flow-mediated dilatation following brachial artery occlusion) on multivariable analysis, potentially supporting the hypothesis of systemic endothelial dysfunction.

### LV systolic dysfunction

Cardiomyocyte dysfunction is recognised in HFpEF. In a large study (*n* = 2042), Borlaug et al. [50] compared echocardiographic load-independent measures (Fig. 2) of systolic function between HFpEF (defined clinically based on Framingham diagnostic criteria), healthy and hypertensive controls. In comparison with healthy controls, mid-wall fractional shortening, a measure of myocardial contractility used to negate the effect of cross-fibre shortening and radial-axis thickening in concentric hypertrophy, was shown to be significantly increased in patients with hypertension without HFpEF but decreased in patients with HFpEF. The difference remained after correcting for afterload and was seen despite similar increases in end-systolic elastance (*E*_ES_), a reflection of systolic workload and stiffness measured as the slope of the end-systolic pressure–volume relationship (ESPVR), thus highlighting the presence of contractile abnormalities despite preserved EF and *E*_ES_ [51]. Similarly, Kraigher-Krainer et al. [52] demonstrated reduced circumferential and global longitudinal LV strains (GLS), measured using speckle-tracking echocardiography, in patients with HFpEF compared with each of healthy and hypertensive controls. GLS was independently associated with natriuretic peptide levels in multivariable analysis but did not associate with *e*ʹ (both septal and lateral measures), *E*/*e*ʹ ratio (septal), or indexed LA volume, suggesting systolic dysfunction as a mechanistic driver of HFpEF independent of diastolic function. In a substudy from the TOPCAT trial, impaired GLS was common (52%) and the strongest echocardiographic predictor of the primary outcome (cardiovascular death, HF hospitalisation or aborted cardiac arrest) with an adjusted HR 1.14 [confidence interval: 1.04–1.24] per 1% decrease on multivariable analysis [53].

Compared with age-matched healthy controls, Tan et al. [54] demonstrated reduced and delayed LV untwisting, both at rest and on exercise, in a small HFpEF cohort, without a significant difference in LV mass or wall thickness. Findings regarding LV twist in HFpEF are conflicting. Tan et al. [54] reported reduced apical rotation, the main determinant of LV twist in patients with HFpEF, compared with healthy controls, and Mordi et al. [35] demonstrated reduced LV torsion in HFpEF; however, other studies have not replicated these findings [55]. Further work is required to understand the utility of twist indices.

### Atrial dysfunction

LA dysfunction is rarely seen without LV diastolic dysfunction and is strongly associated with progressive severity; however, the origin and independent relevance of atrial dysfunction in HFpEF remains contentious [56]. Telles et al. [28] recently demonstrated an inverse correlation between LA reservoir strain **(**Fig. 2**)** and exercise PCWP in HFpEF (*r* = − 0.64, *p* < 0.001), highlighting its potential utility as a marker of dynamic LAP elevation. Using speckle-tracking echocardiography in 308 patients with HFpEF, Freed et al. [57] found an independent relationship between reduced LA reservoir strain and adverse outcomes. As a potential mechanism, LA reservoir strain was associated with markers of pulmonary vascular dysfunction. LA reservoir strain can be used to calculate LA stiffness index (either as PCWP to LA reservoir strain ratio, or as *E*/*e*ʹ to LA reservoir strain ratio), which may be a more discriminative prognostic measure [58]. The multivariable models described by Freed et al. [57] that include LA stiffness index demonstrate a HR 1.39 to 1.44 per standard deviation decrement for the combined outcome of cardiovascular hospitalisation, HF hospitalisation or death. Furthermore, the association remained when the model was adjusted for GLS, suggesting that the impact LA stiffness has on prognosis may be independent of LV function. In a group of 46 patients undergoing surgery for severe mitral regurgitation, Cemeli et al. [59] showed a strong inverse correlation between peak LA reservoir strain and LA fibrosis on histology (*r* = − 0.82, *p* < 0.0001). LA fibrotic burden has been estimated using CMR late-gadolinium enhancement in multiple AF studies; however, the technique is challenging and there is a paucity of histological validation. It is yet to be applied in HFpEF.

### Central and peripheral vascular dysfunction

**Fig. 6:**
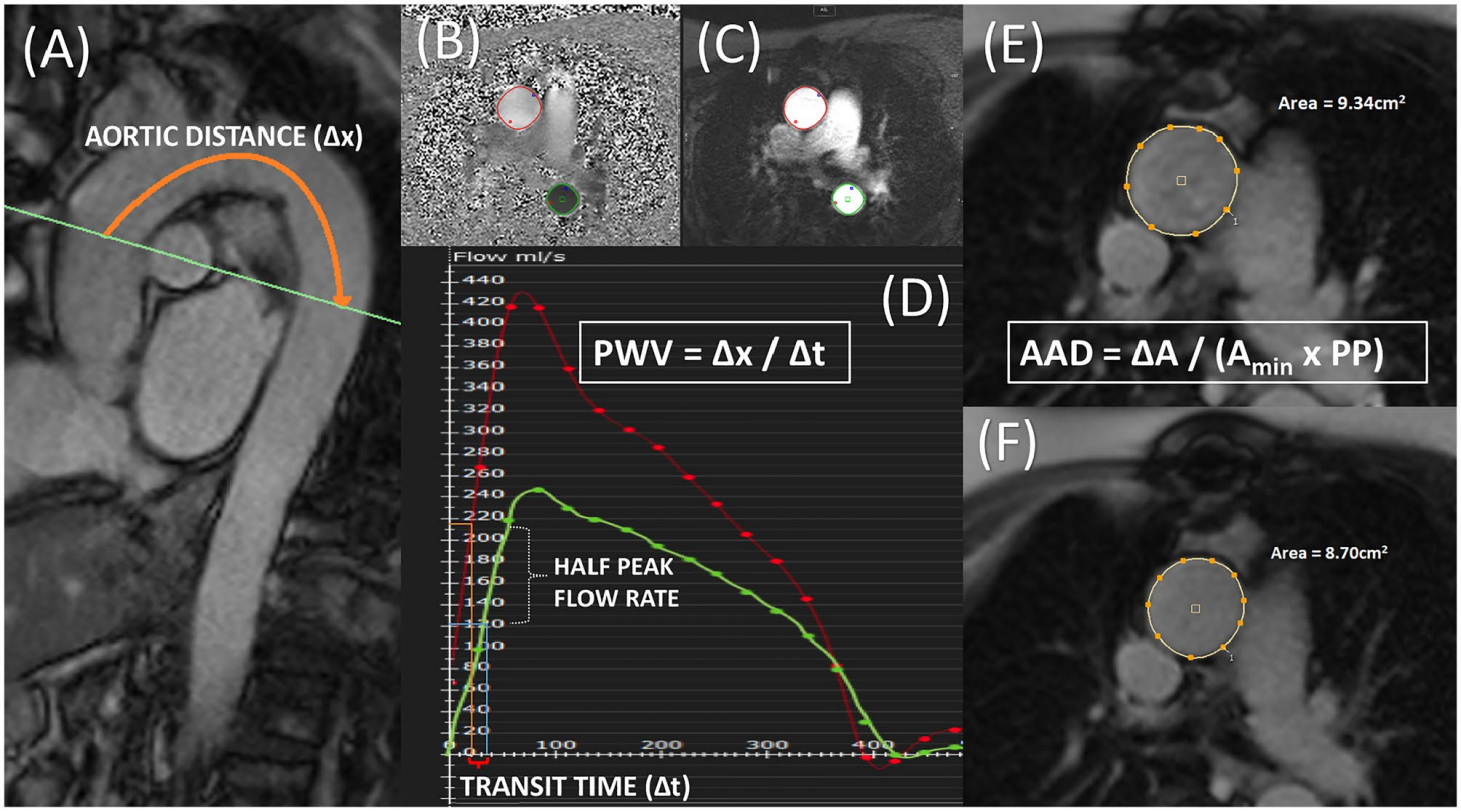
Cardiac magnetic resonance imaging (CMR) measures of arterial stiffness. **a**–**d** Pulse wave velocity (PWV) is calculated between the ascending and descending aorta by the transit-time method. A Aortic ‘candy-stick’ cine is used to plan a through-plane velocity flow map, demonstrated by the green line bisecting the pulmonary artery. The aortic distance between the two aortic locations is measured (∆x). **b**, **c** Through-plane flow and magnitude images generated from the aortic candy stick. The ascending aorta is contoured in Red and the descending aorta in Green. **d** Graph demonstrating flow in the ascending aorta (Red) and descending aorta (Green) during systole. Time to half the peak flow rate is measured and the difference calculated as the transit time (∆*t*). PWV is calculated by the equation PWV = ∆*x*/∆*t*. **e**, **f** Through-plane cine image of the ascending aorta. The maximal (**e**) and minimal (**f**) aortic areas through the cardiac cycle are calculated. Ascending aortic distensibility (AAD) is calculated by the equation AAD = ∆*A* / (*A*_min_ × PP), where ∆*A* represents the change in aortic area, *A*_min_ the minimal aortic area, and PP the pulse pressure

Manifestations of increased arterial stiffness, such as increased pulse wave velocity (PWV), reduced aortic distensibility and increased pulse-wave reflections (PWR), have all been demonstrated in HFpEF **(**Fig. 6**)** [60, 61]. Arterial stiffness is multifactorial, caused by factors such as endothelial dysfunction and reduced nitric oxide bioavailability, increased collagen deposition and accumulation of advanced glycation end products and matrix proteins [62, 63]. A number of imaging modalities have been employed to assess arterial stiffness in HFpEF, including high-frequency ultrasound and CMR [64]. CMR generates cross-sectional aortic cine imaging with high spatial resolution allowing accurate measurement of aortic distensibility [60, 65]. Furthermore, velocity-encoded flow mapping can accurately measure PWV, for which several methods are described (including transit-time, flow-area and cross-correlation methods) [66]. PWR are naturally occurring pressure waves reflected back to the LV at sites of high impedance such as arterial branches. Arterial stiffening leads to faster wave reflections, which arrive back at the LV earlier during systole, leading to increased LV loading in mid-to-late systole, which in turn results in increased systolic workload [61, 67]. Weber et al. [61] demonstrated increased PWR in HFpEF compared with non-HFpEF groups. Compared with hypertensive controls, Reddy et al. [68] showed similar resting PWR in patients with HFpEF; however, these became elevated during exercise. Chirinos et al. [69] showed significantly increased PWV (measured by applanation tonometry) in diabetic HFpEF patients compared to non-diabetic HFpEF patients. Arterial stiffness and increased systemic vascular resistance strongly correlate to exercise limitation and reduced peak oxygen consumption rate (VO_2_) in patients with HFpEF compared with age- and sex-matched healthy controls; however, population studies have failed to demonstrate an independent association between arterial stiffness and incident HFpEF [60, 64, 70, 71].

No change in aortic distensibility was found after 12 months of enalapril or 9 months of spironolactone, compared with placebo, in two randomised controlled trials of HFpEF [65, 72]. Sacubitril/valsartan, which combines anti-fibrotic, anti-inflammatory and vasodilatory effects, and thus may target multiple aspects of arterial dysfunction, could be hypothesised to be more efficacious.

## Risk stratification

Cardiac imaging, in conjunction with clinical indices, has the potential to more precisely risk stratify patients. Shah et al. [24] identified three distinct HFpEF clinical phenotypes with markedly differing prognosis. The first group, which was relatively young (mean age 61 years) and had comparatively low natriuretic peptide levels (median brain natriuretic peptide (BNP) 72 pg/mL) and normal or only mildly abnormal diastolic function, had the best prognosis. The second group had features in keeping with what has previously been described as ‘metabolic HF’, or more recently, the ‘HFpEF obesity phenotype’, with a high prevalence of obesity, diabetes and obstructive sleep apnoea [3, 73]. Diastolic dysfunction (three-quarters had moderate or severe dysfunction), LA dilatation and LV hypertrophy were prominent and right atrial pressure was also relatively high, thought to reflect pericardial constraint, possibly by epicardial adipose tissue. The unadjusted HR for death in this group, in comparison with the first group, was 4.0 [confidence interval: 1.5–10.9]. After adjusting for BNP and the Meta-Analysis Global Group in Chronic Heart Failure (MAGGIC) risk score, the HR for death was 2.2 [confidence interval: 0.8–6.0], which was highest of all of the groups. The third group had the highest prevalence of hypertension and AF and demonstrated the largest LA volumes, highest LV mass, highest BNP levels and most severe diastolic dysfunction. Patients were also more likely to have pulmonary hypertension and right heart dysfunction. In comparison with the first group, the third group had an unadjusted HR for death of 6.5 [confidence interval: 2.5–16.6].

## Discussion

The diagnosis of HFpEF is complex and inconsistent imaging measurements lead to greater ambiguity. Indices of diastolic function are very much in keeping with the ethos of ‘measuring what we can measure, rather than what we want to measure’. Indeed, no echocardiographic index is specific to any particular component of diastolic function, and all are confounded by factors unrelated to diastolic function [6]. The ‘gold-standard’ diagnostic parameter remains resting and exercise invasive LAP measurement. In the setting of normal *resting* echocardiographic indices and natriuretic peptide levels, exercise echocardiographic indices are useful for distinguishing patients that truly have dynamic LAP elevation; however, the technique is far from perfect and current measurements (e.g. *E*/*e*ʹ) may generate a high rate of false-positive results. Assessment of diastolic dysfunction during exercise requires further refinement.

As a result of the limitations of current measurements of diastolic function, HF symptoms may be wrongly classed (or ‘dismissed’) as ‘non-cardiac’ [23, 24, 26, 28, 58]. Furthermore, mandating that particular structural abnormalities, such as LA dilatation and increased LV wall thickness, be present for a diagnosis of HFpEF, as is the case in recent and on-going trials, means that some patients with confirmed HFpEF are excluded from such trials [4, 23, 74, 75].

So, what for the future? Whilst, as discussed, a number of imaging measurements hold promise for more meaningful assessment of diastolic function, there is no non-invasive index on the horizon that seems likely to overcome the current limitations. But perhaps that is the wrong approach in any case. Having identified a patient with symptoms ± signs of HF, raised natriuretic peptide levels and a preserved LVEF, perhaps the next step in the diagnostic pathway should be to search for the underlying disease mechanism, for it is this that an intervention would aim to target, and it is this that will determine prognosis. Does the patient have fibrotic-HFpEF, do they have arterial dysfunction-HFpEF, do they have atrial dysfunction-HFpEF? We currently do not routinely investigate for the cause of HF in the context of a preserved EF, and as such, it is entirely unsurprising that we do not have a treatment. Indeed, as an analogy, we currently stop at ‘anaemia’, rather than going on to determine the cause of the anaemia and hence the underlying diagnosis. HFpEF is not a diagnosis.

Imaging can help. As described, contemporary imaging techniques can delve into the myocardium and identify those patients with myocardial fibrosis, with cellular hypertrophy, with impaired energetics, and assess the wider cardiovascular system to identify those patients with atrial dysfunction and with arterial dysfunction. Myocardial fibrotic-HFpEF is a good example. Myocardial fibrosis can be measured non-invasively with CMR and myocardial fibrosis is strongly associated with prognosis in HFpEF. If we routinely assessed for myocardial fibrosis, we could potentially target those patients displaying it with antifibrotic interventions, as is under investigation in the ongoing PIROUETTE trial (NCT02932566) [42].

Reclassifying the complexity of HFpEF into more appropriate diagnoses that take into account underlying disease mechanisms and clinical factors is likely to require techniques such as machine learning, as used by Shah et al. and others. Such techniques allow “agnostic’ variable selection, in which optimal groups of variables are chosen through iterative statistical modelling, rather through human endeavour [76]. Grouping together pathophysiologically similar individuals would provide the basis for developing and evaluating tailored therapy, provide patients with more appropriate diagnoses, allow personalised risk stratification and better guide patient care.
